# Rotaviruses and Rotavirus Vaccines

**DOI:** 10.3390/pathogens10080959

**Published:** 2021-07-29

**Authors:** Celeste M. Donato, Julie E. Bines

**Affiliations:** 1 Enteric Diseases Group, Murdoch Children’s Research Institute, Parkville, VIC 3052, Australia; jebines@unimelb.edu.au; 2 Department of Paediatrics, The University of Melbourne, Parkville, VIC 3052, Australia; 3 Department of Microbiology, Biomedicine Discovery Institute, Monash University, Clayton, VIC 3800, Australia; 4 Department of Gastroenterology and Clinical Nutrition, Royal Children’s Hospital, Parkville, VIC 3052, Australia

**Keywords:** epidemiology, rotavirus vaccines, molecular phylogeny, virus evolution, zoonosis, vaccines, virology

Group A rotaviruses belong to the *Reoviridae* virus family and are classified into G and P genotypes based on the outer capsid proteins VP7 and VP4, respectively. To date, 36 G types and 51 P types have been characterised from humans and varied animal species [1]. The most prevalent genotypes in humans are G1, G2, G3, G4, G9, and G12, in combination with P[4], P[6], and P[8] [2,3]. A whole genome classification nomenclature has been developed to describe the genome constellation of strains; Gx-P[x]-Ix-Rx-Cx-Mx-Ax-Nx-Tx-Ex-Hx, denoting the VP7-VP4-VP6-VP1-VP2-VP3-NSP1-NSP2-NSP3-NSP4-NSP5/6 genes, with x referring to the various recognised genotypes for each gene. There are three major genotype constellations: Wa-like (G1-P[8]-I1-R1-C1-M1-A1-N1-T1-E1-H1), DS-1-like (G2-P[4]-I2-R2-C2-M2-A2-N2-T2-E2-H2), and AU-1-like (G3-P[9]-I3-R3-C3-M3-A3-N3-T3-E3-H3) [4].

Group A rotaviruses remain one of the principal aetiological agents of acute gastroenteritis in infants and young children worldwide. Rotavirus infection was estimated to have caused 128,500 deaths (95% uncertainty interval (UI), 104,500–155,600) and 258,173,300 episodes (95% UI, 193 million to 341 million) of diarrhea among children under 5 years of age in 2016 [5]. Rotavirus-associated mortality rates are highest in sub-Saharan Africa, Southeast Asia, and South Asia [5]. There has been a substantial decrease in the global burden of rotavirus disease over the last decade which can be attributed to varied public health measures such as improved sanitation, as well as the inclusion of rotavirus vaccines into the National Immunisation Programs of over 112 countries worldwide [6]. The implementation of rotavirus vaccines has been estimated to have averted more than 28,000 deaths (95% UI, 14,600–46,700) among children under 5 years of age in 2016 [5]. However, many low- and middle-income countries are yet to introduce rotavirus vaccines. The expanded use of the rotavirus vaccines, particularly in sub-Saharan Africa, could have prevented approximately 20% of all deaths attributable to diarrhea among children under 5 years of age in 2016 [5].

Four group A rotavirus vaccines; Rotarix^®^ (GlaxoSmithKline, Rixenstart, Belgium), Rotasiil^®^ (Serum Institute of India, Pune, India), RotaTeq^®^ (Merck & Co, Pennsylvania, PA, USA) and Rotavac^®^ (Bharat Biotech, Hyderabad, India) have been prequalified by the World Health Organization (WHO) for global use [7]. The most widely used vaccines are Rotarix, which is a monovalent vaccine comprised of a single human G1P[8] strain and RotaTeq, which is a pentavalent, human-bovine reassortant vaccine comprising G1P[5], G2P[5], G3P[5], G4P[5] and G6P[8] strains [8,9]. Rotasiil and Rotavac are primarily used in India. Rotasiil is a pentavalent, human-bovine reassortant vaccine comprised of G1P[5], G2P[5], G3P[5], G4P[5], and G9[5] [10]. Rotavac is a monovalent vaccine comprised of a naturally occurring G9P[11] reassortant strain [11].

The goal of this special edition of *Pathogens* was to bring together a breadth of information on rotavirus and rotavirus vaccines globally to highlight rotavirus research from across the world that represent recent advances in our knowledge of vaccine effectiveness, rotavirus epidemiology, genotypic diversity, and genomic characterisation.

High rates of vaccine effectiveness have been reported in Europe, the USA and Australia [12,13]. However, suboptimal vaccine effectiveness, especially in the second year of life, has been noted in low- and middle-income countries in Africa and Latin America, as well as during outbreaks of rotavirus disease in the Australian Indigenous population [14,15]. The reasons why vaccine take and subsequent vaccine effectiveness are lower in some settings remains unclear. A range of factors have been implicated including higher rotavirus transmission rates, variations in gut microbiota, and host factors such as histo-blood group antigen (HBGA) and Lewis secretor antigens [15]. Middleton et al. conducted a retrospective case–control study to evaluate the performance of Rotarix and RotaTeq during a G2P[4] rotavirus epidemic in rural and remote Australia [16]. The majority of affected children were Aboriginal and/or Torres Strait Islander; populations that experience a disproportionately high burden of rotavirus disease. During this G2P[4] outbreak, there was some evidence of a protective effect among younger children under 12 months of age. However, the overall protective effect of either Rotarix or RotaTeq in this setting was weak. The study highlights that even within a high-income country, certain populations will experience differing vaccine effectiveness, which suggests that tailored vaccine strategies and public health measures may be required to better protect these populations until the reasons for suboptimal vaccine effectiveness can be elucidated and addressed [16]. The strain associated with this outbreak was characterised by Donato et al. and the demographics of the outbreak in Kimberley region of Western Australia was described [17]. Full genome sequencing revealed the outbreak variant exhibited the archetypal DS-1-like genome constellation: G2-P[4]-I2-R2-C2-M2-A2-N2-T2-E2-H2 and phylogenetic analysis revealed all genes were closely related to contemporary Japanese G2P[4] samples indicating a recent introduction into Australia rather than the outbreak variant having been derived from G2P[4] variants that had caused prior outbreaks in the region. The VP7 gene of the outbreak variant was compared to the G2 component of the RotaTeq vaccine, identifying mutations in known antigenic regions. However, these mutations are frequently observed in contemporary G2P[4] strains and are unlikely to be the sole reason that the outbreak occurred this population [17].

Host genetic factors such as HBGA status play a role in susceptibility to disease as well as response to vaccines. HBGA status varies across populations; with higher proportions of non-secretor phenotypes observed in African populations compared to other populations [18]. MacDonald et al. investigated FUT2-defined secretor status in children under 5 years-old hospitalised with rotavirus-related diarrhoea compared with rotavirus-negative controls [19]. The proportion of secretors in rotavirus-positive cases was significantly higher than in the rotavirus-negative controls. The rotavirus genotypes P[8] and P[4] were detected at significantly higher proportions in secretors compared to non-secretors. However, the P[6] genotype was observed at similar proportions amongst secretor and non-secretors [19]. Overall, this study suggests that HGBA status may partially influence rotavirus infection due to the VP4 protein, and may explain why rotavirus vaccines with P[8] strains exhibit suboptimal effectiveness in African populations [19].

Numerous mechanisms including genetic drift and reassortment contribute to rotavirus diversity. The segmented genome allows for reassortment both within and between human and animal strains, leading to the emergence of novel strains and unusual genotype combinations [20]. Reassortment is a key mechanism driving the evolution of rotavirus strains and reassortment between strains of different genotypes have been increasingly observed. The emergence of DS-1-like G1P[8] strains have been reported in several countries during the rotavirus vaccination era [21]. Mwangi et al. reported atypical DS-1-like G1P[8] strains that circulated in 2008 during the pre-vaccine era in South Africa [22]. These strains emerged through reassortment events involving locally circulating South African strains and were not related to other atypical G1P[8] strains reported globally. This study highlighted the occurrence of independent, local reassortant events contributing to rotavirus diversity [22].

Zoonotic transmission also plays a critical role in the diversity of rotavirus strains detected in the human population. Maringa et al. described the full genome constellation of a human-porcine reassortant strain, RVA/Human-wt/ZMB/UFS-NGS-MRC-DPRU4723/2014/G5P[6], which was identified from an unvaccinated 12 month old male who had been hospitalised for gastroenteritis in Zambia [23]. The strain exhibited the genome constellation G5-P[6]-I1-R1-C1-M1-A8-N1-T1-E1-H1 and phylogenetic analysis revealed the genes were most closely related to porcine and porcine-like human strains [23]. Understanding the diversity of strains in various animal populations is important for animal health and farming practices, as well as being informative for the contextualisation of zoonotic transmission events. Castells et al. reported detection of rotavirus in calves reared for beef and dairy production in Uruguay [24]. Multiple genotypes associated with bovine disease were detected including G6P[11] (40.4%), G6P[5] (38.6%), G10P[11] (19.3%), as well as the uncommon genotype G24P[33] (1.8%) [24].

Long-term surveillance is critical in understanding the trends in rotavirus genotype diversity and distribution, as overinterpretation of short-term fluctuations in genotype prevalence can be misleading with without a greater context of the natural, cyclic patterns in genotype replacement over time. This is especially critical when comparing trends in genotype distribution pre- and post-vaccine introduction. Genotype surveillance data in Australia and elsewhere has revealed changes in diversity, as well as temporal and geographic fluctuations over time following vaccine introduction [25,26]. Furthermore, differences in genotype diversity and dominance were observed when comparing vaccines by jurisdictions, suggesting that RotaTeq and Rotarix may exert different immunological pressures [25].

Yandle et al. described changes in the burden of rotavirus disease and genotype distribution in Ireland following vaccine introduction [27]. Rotavirus detection decreased by 91% in children aged 0–12 months between 2015/16 and 2018/19, and the once prominent seasonal peak in disease was reduced following Rotarix vaccine introduction in December 2016. The genotype distribution altered following vaccine introduction; the prevalence of G1P[8] which was dominant prior to vaccine introduction decreased while the prevalence of G2P[4] and G3P[8] increased. An increase in genotype diversity was also observed in the vaccine era, with the equine-like G3P[8] variant detected in Ireland [27]. The equine-like G3P[8] variant was also reported by Gutierrez et al., where it was the dominant genotype observed in Brazil in 2018 and 2019 [28]. This study also reported a significantly higher positivity rate among children aged >24–60 months compared to other age groups [28]. This shift in the age of rotavirus disease towards slightly older children has also been reported in other countries that have introduced rotavirus vaccines [26,29]. The equine-like G3P[8] variant was also reported by Mwanga et al., from the Kilifi region of Kenya in 2018; four years after Rotarix was introduced [30]. During this surveillance year, G3P[8] was the dominant genotype detected and the equine variant accounted for a small proportion of these G3P[8] cases, replacing G2P[4] and G1P[8] which had been predominant in the prior two years [30]. The epidemiological trends of enteric viruses pre- and post-rotavirus vaccine introduction were investigated in this region by Lambisia et al., describing rotavirus, norovirus (genogroup GII), adenovirus, astrovirus and sapovirus [31]. Following the introduction of Rotarix, the prevalence of rotavirus decreased whilst the prevalence of norovirus increased. The prevalence of adenovirus, astrovirus and sapovirus remained unchanged. This study also reported an increase in the median age of diarrhoea cases [31].

Rotarix was introduced into the Fiji National Immunisation Program in 2012 and has reduced the burden of rotavirus disease and hospitalisations in children under 5 years of age. Ongoing rotavirus surveillance has been conducted in Fiji to investigate changes in genotype diversity, and Thomas et al. described patterns of rotavirus genotype diversity from 2005 to 2018 [32]. Prior to vaccine introduction, genotype dominance fluctuated annually and G1P[8] and G2P[4] were the dominant genotypes. In contrast to many countries that have reported an increase in rotavirus genotype diversity in the vaccine era, a decrease in diversity was observed in Fiji. G1P[8] and G2P[4] were not detected after 2015 and 2014, respectively. Similar to reports from Australia, G3P[8] and G12P[8] were frequently detected in the vaccine-era and the equine-like G3P[8] variants was transiently detected in between 2015–2016 [25,32].

Long-term surveillance pre- and post-vaccine introduction was also conducted at five sentinel sites in India between 2012 to 2020 by Varghese and colleagues [33]. The Rotavac vaccine was introduced in 2016 resulting in a decrease in rotavirus-associated hospitalisations. G1P[8] was the predominant genotyped reported in the pre-vaccination period, whereas G3P[8] became the dominant genotype in the post-vaccination period. Geographic variation in genotype distribution was noted between northern and southern sites [33].

João et al. also reported the prevalence of rotavirus genotypes, pre- (2012–2015) and post-vaccine (2016–2019) introduction in Mozambique [34]. In the three years prior to Rotarix vaccine introduction, G9P[8] was the predominant genotype with G1P[8], G2P[4] and G12P[4] also frequently detected. Following vaccine introduction G1P[8] remained a predominant genotype which is unusual as the prevalence of G1P[8] has been reported to dramatically decrease in most vaccine settings. The prevalence of G9P[8], G2P[4] and G12P[4] decreased while G9P[4] and G3P[4] emerged as prevalent genotypes [34]. A companion study from Munlela et al. described the whole genome characterisation and evolutionary analysis of Mozambican G1P[8] strains pre- and post-vaccine introduction [35]. The strains were collected between 2012 and 2017 and all exhibited a Wa-like genome constellation (G1-P[8]-I1-R1-C1-M1-A1-N1-T1-E1-H1). Phylogenetic analysis revealed the majority of strains clustered closely together in a conserved clade across the entire genome. No distinct clustering for pre- and post-vaccine strains were observed and strains appeared to have been derived from multiple introductions into Mozambique, potentially from India due to the high degree of genetic similarity across the genome. There was no discernible vaccine-induced selection pressure observed in this study [35].

The constant alternations in rotavirus genotype diversity and prevalence within countries and globally highlights the importance of on-going epidemiological and molecular surveillance programs. The increasing detection of unusual zoonotic and reassortant strains emphasises the necessity of whole genome sequencing and detailed phylogenetic analysis. Concerns remain that widespread vaccine use may shape the diversity of rotavirus strains and that vaccine-escape variants may emerge in some settings. Understanding the long-term patterns of rotavirus genotype distribution and evolution is critical in order to assess any changes observed following vaccine introduction given the tendency for natural temporal and geographic fluctuations in the absence of vaccines.

The continued surveillance and characterisation of rotavirus genotypes circulating in the vaccine era globally will provide important insights into epidemiology and strain diversity, ensuring the success of current and future vaccination programs. We express our sincere thanks to the authors and reviewers for their contribution to this very important topic. We also express our sincere gratitude to the Bill and Melinda Gates Foundation for providing funds to support the publication fees for the articles in this special edition.
